# Geostatistical characterization of the soil of Aguascalientes, México, by using spatial estimation techniques

**DOI:** 10.1186/s40064-016-2593-7

**Published:** 2016-06-29

**Authors:** Ricardo Magdaleno-Márquez, María de la Luz Pérez-Rea, Víctor M. Castaño

**Affiliations:** División de Estudios de Posgrado, Facultad de Ingeniería, Universidad Autónoma de Querétaro, Cerro de las Campanas s/n, Col. Niños Héroes, 76010 Querétaro, QRO Mexico; Centro de Física Aplicada y Tecnología Avanzada, Universidad Nacional Autónoma de México, Boulevard Juriquilla, 3001 Santiago De Querétaro, QRO Mexico

**Keywords:** Spatial interpolation, Splines, Standard Kriging, Geostatistics, Geographical infromation systems, Aguascalientes

## Abstract

Four spatial estimation techniques available in commercial computational packages are evaluated and compared, namely: regularized splines interpolation, tension splines interpolation, inverse distance weighted interpolation, and ordinary Kriging estimation, in order to establish the best representation for the shallow stratigraphic configuration in the city of Aguascalientes, in Central Mexico. Data from 478 sample points along with the software ArcGIS (Environmental Systems Research Institute, Inc. (ESRI), ArcGIS, ver. 9.3, Redlands, California 2008) to calculate the spatial estimates. Each technique was evaluated based on the root mean square error, calculated from a validation between the generated estimates and measured data from 64 sample points which were not used in the spatial estimation process. The present study shows that, for the estimation of the hard-soil layer, ordinary Kriging offered the best performance among the evaluated techniques.

## Background

The city of Aguascalientes, in a state of the same name, in central Mexico, is nestled in a valley alluvial (Aranda-Gómez and Carrillo 1983; Aranda-Gómez and Aranda-Gómez 1985), so subsoil consists of a mixture of coarse and fine soils, the latter being posed a challenge to builders (Arroyo-Contreras et al. 2004). In the Valley of Aguascalientes the problem is the presence of surface deposits collapsible settlements and causing damage to structures.

Geographic information systems are effective tools for geotechnical engineering as all information used has spatial attributes (Player 2006), thus facilitating the implementation of spatial interpolation techniques and geostatistics for estimating soil properties as the collapsible layer thickness surface. There are a wide range of spatial estimation techniques, but not easy to determine which one will yield better results as the phenomenon to be studied.

In the scientific literature has tried to resolve this issue, using two basic methods. The first of these studies consists of reconciliation, or to compare the results of the estimation results with different methods of production (Healey 1993; Sides 1992). The other method is to simulate a sector, testing different estimation techniques and compare the results with the simulated reality (Bell and Whateley 1994).

The objective of this study is to evaluate and compare four spatial estimation techniques: the regularized spline interpolation, by splines in tension, by the inverse distance weighted and ordinary Kriging estimation to determine which best suits the surface stratigraphy settings in the city of Aguascalientes.

### Geographical location

**Fig. 1:**
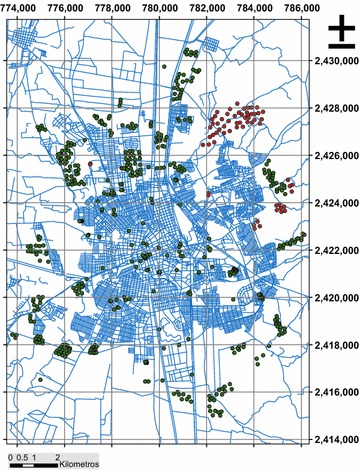
Geographic location of the city of Aguascalientes and location of the probes, the* green dots* correspond to the 478 surveys used in the estimation, and* orange dots* at 64 probes used for the assessment of the estimates

The city of Aguascalientes is located between the extreme projected coordinate X1 = X2 = 2416000 and 2430000 north latitude, and between Y1 = Y2 = 774.000 and 786.000 west longitude coordinate system Universal Transverse Mercator, North American Datum of 1927 (UTM-NAD27, Zone 13 North) (Fig. 1), with an average elevation of 1870 m above mean sea level, nestled in the Valley of Aguascalientes which is a morphological subunit crosses the east-central part of the state, with an orientation substantially NS, and that an elongated depression is between 10 and 20 km. wide, extending beyond state boundaries, from south of Zacatecas to the eastern end of Jalisco (Arroyo-Contreras et al. 2004). Depression is an alluvial plain formed by more or less regular reaching an altitude between 1850 and 1900 m levels, where the river runs central Aguascalientes (also known as San Pedro River), which flows from north to south and is the most important state flow.

The Valle de Aguascalientes corresponds to a tectonic graben structure, known in geological literature as the “Graben of Aguascalientes”, bounded on the east and west by two large faults oriented north–south, this is not no rock formation emerges as filling materials are unconsolidated alluvial deposited on a bedrock these fillers thickness reaches more than 600 m. in the deeper parts and not less than 200 m. in the more shallow (Aranda-Gómez and Aranda-Gómez 1985; Arroyo-Contreras et al. 2004).

From 1979 cracks were detected in the urban area of the city of Aguascalientes and other towns in the surrounding area as Jesus Mary and Puertecito Chicalote of the Virgin, all located in the Quaternary alluvial fill (Aranda-Gómez and Aranda-Gómez 1985). The direction of these cracks coincides with the general direction of the valley with maximum vertical displacement of 1 m or more. Invariably, with few exceptions, the downthrown the crack is located to the west of the city, that is, toward the San Pedro River. On the eastern edge of the city is located a normal fault with approximately NS direction, which is considered that originated the Graben of Aguascalientes, from this failure, the surface of the city has an east–west slope greater than 1 %.

## Methods

The computer software ArcGIS (ESRI 2008) was used to implement the spatial estimation techniques by regularized splines, Splines in tension, weighted inverse distance and ordinary kriging estimation, and determine which one best represents the stratigraphy surface conditions of the city of Aguascalientes.

The data used in estimating the records consist of 478 polls open pit type (PCA) performed within the urban area of the city (Fig. 1), which were obtained from the files of the Department of Splits Development Secretariat Aguascalientes urban township, and company Integral Civil Works Supervisor, S. A. de CV, from the same sources were obtained records of 64 polls PCA type (Fig. 1) with additional assessment was made of each technique.

The open pits are scattered all over, with no obvious pattern of the spacing between them, as they were made for particular studies and do not share the same information among each other. The depth of the open holes varies between 3.00 and 5.00 m. Generally speaking, two layers can be identified: a collapsible surface layer composed mainly of clay or silt of low compressibility (CL or ML) and a second resistant layer, with high stiffness, composed by cemented silty sands or clayey materials (SM or SC). The depth of the hard layer is the basic parameter for the analysis. The mechanical properties of the soil obtained from a stratigraphic profile were used to establish the depth of this layer.

The data collected were synthesized in a database created with the coordinates of all the polls and the depth of the hard layer (surface layer thickness) in meters represented negative (−m) to characterize the relief of the hard layer (composed silty sand or gravel-sand). For the location of the probes was used UTM projected coordinate system, with the North American Datum of 1927 (Fig. 1).

The hard layer depth was estimated using four techniques, obtaining for each estimate a raster file which was processed and compared with data from the probes 64 prepared for evaluation, using the calculation of the root mean square error (RMSE) to evaluate and compare each technique. RMSE was chosen as a benchmark as it retains the same units as the input data.

### Spatial estimation techniques

The hard layer depth was estimated using the four techniques mentioned above. Since the base of each appears widely described in the literature (Isaaks and Srivastava 1989; Armstrong 1998; Webster and Oliver 2001), only given general elements each.

#### Regular splines and splines in tension

These are techniques which estimate deterministic interpolation values using a function that minimizes the overall curvature of the surface, resulting in a smooth surface that passes exactly through all known points of entry.

The basic shape of the spline interpolation imposes two conditions on the interpolation function:

1. The surface must pass exactly through the input data.
2. The surface must have a minimum curvature: the sum of the squares of the second derivatives calculated at each point of the surface should be as short as possible.

Splines There are two techniques: the regularized and the voltage in the regularized creates a smooth, gradual change with values that may be generated outside the range of the data sample, and the tensile stiffness is controlled surface according to the nature of the phenomenon to model, it creates a smooth surface with values less linked to the range of the sample data.

Splines regularized technique modifies the criterion of minimizing the curvature, incorporating the third derivative and assigning a weight, referred to as τ in the literature. This method is especially useful if required the calculation of the second derivatives of the surface of interpolation.

Splines technique used in tension arising in the first criterion of minimizing the curvature, assigning a weighting referred to as φ in the literature. The generated surface is continuous and smooth, while the first derivative is continuous, but is not smooth.

Both techniques use the following expression Splines to generate surface interpolation:

1 $$ S\left( {x,y} \right) = T\left( {x,y} \right) + \mathop \sum \limits_{j = 1}^{N} \lambda_{j}  R\left( {r_{j} } \right) $$

where ***N***, is the number of input points, *λ*_*j*_ are the weight coefficients and *r*_*j*_ is the distance between the point to be estimated *(x, y)* and the *j input point.*

*T* (*x*, *y*) y *R* (*r*_*j*_) are defined according to the specific Spline used, as follows:for the regular splines:

2 $$ T\left( {x,y} \right) = a_{1} + a_{2} x + a_{3} y $$

3 $$ R\left( r \right) = \frac{1}{2\pi } \left\{ {\frac{{r^{2} }}{4} \left[ {\ln \left( {\frac{r}{2\tau }} \right) + c - 1} \right] + \tau^{2} \left[ {K_{0} \left( {\frac{r}{\tau }} \right) + c + { \ln }\left( {\frac{r}{2\pi }} \right)} \right]} \right\} $$

for the splines in tension:

4 $$ T\left( {x,y} \right) = a_{1} $$

5 $$ R\left( r \right) = - \frac{1}{{2\pi \varphi^{2} }} \left[ {\ln \left( {\frac{r\varphi }{2}} \right) + c + K_{0} \left( {r\varphi } \right)} \right] $$

where *τ* y *φ* are the weighing parameters, *r* is the distance between the point to be estimated and the input point, *K*_0_ is the modified Bessel function, *c* = 0.577215, and *a*_*i*_ are coefficients of the associated system of linear equations.

#### Weighted inverse of distance

This method implicitly assumes that parts of the data that are nearby are more similar than those further away. To estimate a value at a given point using a linear combination of neighboring data, so it is assumed that each input has a local influence that decreases with distance.

The general term used for this method is:

6 $$ z^{*} \left( {x_{0} } \right) = \mathop \sum \limits_{i = 1}^{N} \lambda_{i}  z(x_{i} ) $$

where *x*_0_ is the point where the estimation is to be made, $z(x_{i} )$, *i* = 1, 2, …, N, are the actual measurements of the point *x*_*i*_, and *λ*_*i*_ are the weight coefficients associated to each measurement; the estimation itself is symbolized by z^*^(*x*_0_).

The weighting coefficients are calculated based on the inverse of the distance between the target point and the locations *x* 0 *xi* input measurements:

7 $$ \lambda_{i} = 1/\left| {x_{i} - x_{0} } \right|^{\beta } \quad  {\text{con}} \beta > 0 $$

The preferred choice for *β* is 2, thus, the estimation corresponds to the square of the inverse of the distance.

The only requirement of this method is that the sum of weights equals λ *i* unit. Thus, if *x* 0 *xi* match then the weight for that point becomes unitary and xz* takes the value 0 *z* (*xi*), so that interpolation is accurate.

An attractive feature of this technique is that the weights decrease rapidly as the distance increases, so that the local estimation is significantly further since the weights never become null no discontinuities. However, the selection of the weighting function is arbitrary, and there is a measure of the estimation error. Furthermore, the created surface always has a zero gradient in the entry points, so that the maxima and minima occur only at those sites.

#### Standard Kriging

Kriging methods rely on mathematical and statistical models, and it is precisely the addition of statistical models which separates deterministic Kriging methods, the Kriging uses or functions of semi-variance or variance as a function of distance, which is characteristic geostatistics distinctive and allows models based on this semi-variance.

The technique of ordinary Kriging is to use estimators that are linear combinations of the data: for point estimates of the variable V (X) at a point x0 we use the estimator *V* * ***x*0**, as a linear combination of known data *V* (*xi*):

8 $$ V^{*} \left( {x_{0} } \right) = \mathop \sum \limits_{i = 1}^{n} \lambda_{i} V(x_{i} ) $$

To ensure that the estimator is unbiased, the sum of the weights must be equal to unity:

$$ \mathop \sum \limits_{i = 1}^{n} \lambda_{i} = 1 $$

And the expected error should be zero:

$$ E\left[ {V\left( X \right) - V^{*} \left( X \right)} \right] = 0 $$

Thus, the variance of the estimate is:

9 $$ Var\left[ {V\left( {x_{0} } \right)} \right] = E\left\{ {\left( {V\left( X \right) - V^{*} \left( X \right)} \right)^{2} } \right\} = 2 \mathop \sum \limits_{i = 1}^{N} \lambda_{i}  \gamma \left( {x_{i} ,x_{0} } \right) - \mathop \sum \limits_{i = 1}^{N} \mathop \sum \limits_{j = 1}^{N} \lambda_{i}  \lambda_{j}  \gamma \left( {x_{i} ,x_{j} } \right) $$

where γ (*xi*, *xj*) is the semi-variance of V (X) between the data points *xi* and *xj*, and γ (*xi*, *x*0) is the semi-variance between the i-th data point and the target point *x* 0, with the following expression for the semi-variance:

10 $$ \gamma (h) = 1/2 E\left\{ {\left[ {V\left( {x_{i} } \right) - V\left( {x_{i} + h} \right)} \right]^{2} } \right\} $$

where h is a scalar equal to the distance between the points and *xjxi*.

For each estimated value exists kriging variance associated, which we denote as σ^2^ ( 0), defined by Eq. (9). The next step is to determine the coefficients which minimize λ *i* variance value, this is done using the method of Lagrange multipliers.

First we define an auxiliary function *f* (λ *i*, ψ) containing that we want to minimize the variance plus a term that contains a multiple of Lagrange, ψ, for ordinary Kriging this function is:

11 $$ f\left( {\lambda_{i} , \psi } \right) = Var\left[ {V\left( {x_{0} } \right) - V^{*} \left( {x_{0} } \right)} \right] - 2\psi \left\{ {\mathop \sum \limits_{i = 1}^{n} \lambda_{i} - 1} \right\} $$

After defining the partial derivatives of the function with respect to the coefficients λ and ψ regarding:

$$ \frac{{\partial f\left( {\lambda_{i} , \psi } \right)}}{{\partial \lambda_{i} }} = 0, $$

$$ \frac{{\partial f\left( {\lambda_{i} , \psi } \right)}}{\partial \psi } = 0, $$

for i = 1, 2,…, N. This leads to a system of N + 1 equations with the same number of unknowns:

12 $$ \mathop \sum \limits_{i = 1}^{N} \lambda_{i}  \gamma \left( {x_{i} ,x_{j} } \right) + \psi \left( {x_{0} } \right) = \gamma \left( {x_{j} ,x_{0} } \right) $$

The solution of this system of equations provides the coefficients λ *i*, which are used in Eq. (8) for the estimates.

### Evaluation and comparison

To compare the results obtained by each technique was used root mean square error (RMSE):

13 $$ {\text{RECM}} = \sqrt {\frac{1}{\text{N}} *\mathop \sum \limits_{i = 1}^{\text{n}} \left( {v_{i}^{*} - v_{i} } \right)^{2} } $$

where N is the number of validation points, and *νi* and νi* are estimated and measured values at each location i.

## Results and discussion

Table 1 shows the basic statistical data of the layer depth.

**Table 1:** Statistical description of depth of hard layer (m)

Media	−0.9323
Median	−0.675
Mode	−0.50
Standard deviation	0.8111
Variance	0.6579
Range	4.75
Minumum	−4.80
Maximum	−0.05
Count	478

**Fig. 2:**
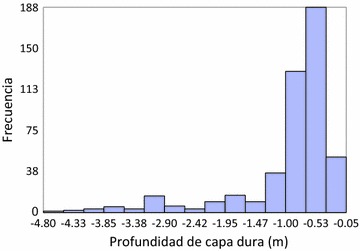
Histogram data hard layer depth

The data distribution is asymmetrical negative as the average is less than the median, having a bias towards the left, it can be seen that the shape of the histogram (Fig. 2).

**Fig. 3:**
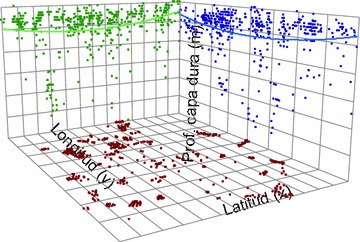
Trend analysis of the data, the* brown spots* show the location of the probes, the* green and blue dots* show the values of hard layer depth, projected against the latitude and longitude respectively, and the lines represent the corresponding fitting functions

We also carried out an analysis of spatial trend, showing a clear trend in the data, which was modeled by a quadratic polynomial fit (Fig. 3). The trend analysis and the analysis of the distribution were useful for the implementation of the estimation by ordinary kriging, since this technique is based on statistical parameters and spatial variability (Isaaks and Srivastava 1989; Webster and Oliver 2001).

**Fig. 4:**
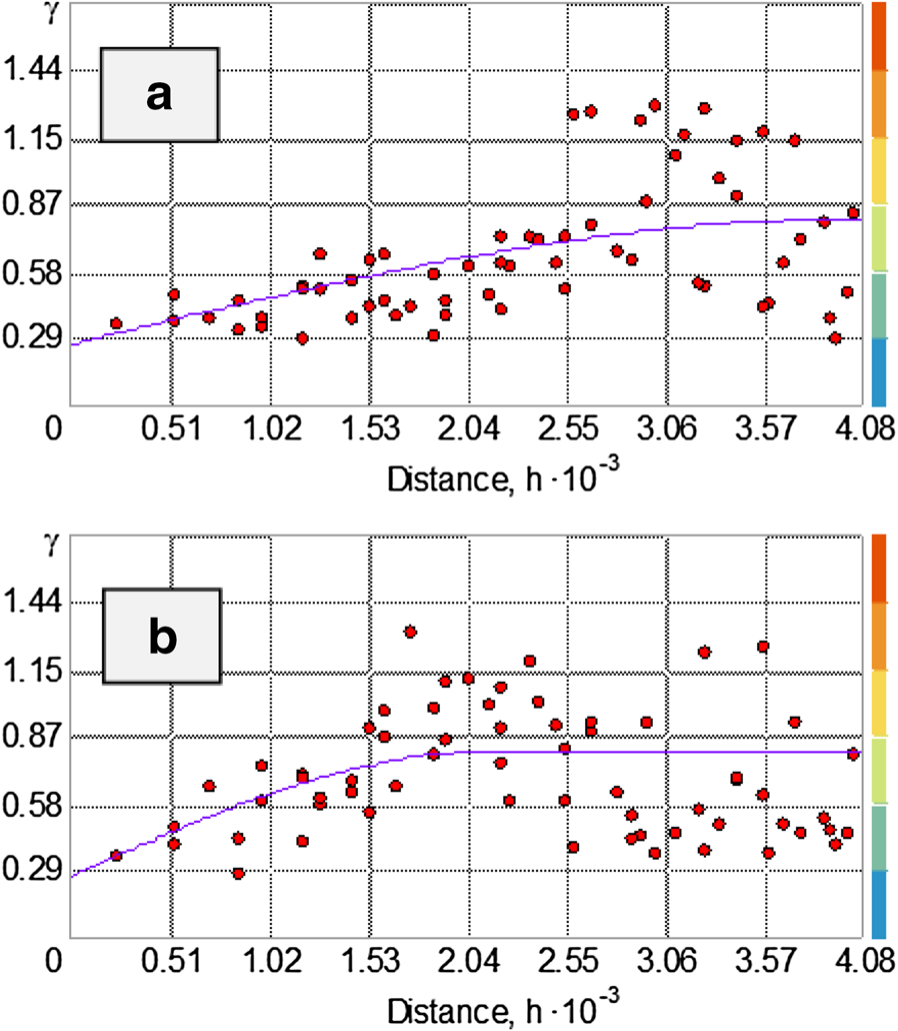
Spherical directional variograms, **a** azimuthal direction 17.10°, **a** 107.10° azimuthal direction

**Fig. 5:**
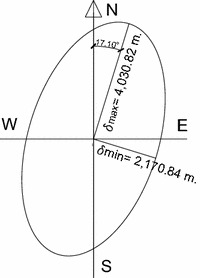
Ellipse of anisotropy

The variograms adjusted (Fig. 4) permitted the identification anisotropy in the spatial variability since not all the directions have the same distance phenomenon correlation, the correlation distance is minimum is in the direction of azimuthal = 107.10° and is 2170.84 m. and the higher correlation distance of 4030.82 m. in the azimuth = 17.10° (Figs. 4, 5) could also capture the variability in distance below the sample, through the “nugget effect” (or “nugget” in English) found 0256.

### Regularized splines

**Fig. 6:**
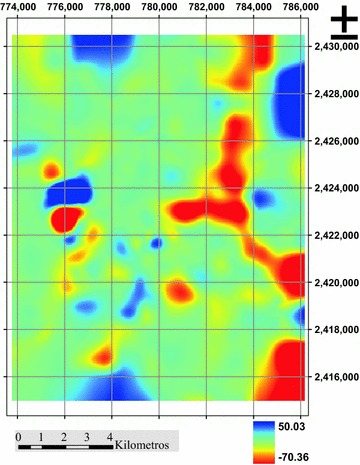
Estimating hardpan depth (m), Splines technique regularized

The estimate generated by this technique presents very erratic values and even positive (Fig. 6), the phenomenon being modeled is hard layer depth in meters negative (−m). As RMSE values, this technique had the highest value of four, with RMSE = 4.937 (Table 2), so that this technique is more yielding inaccurate results.

**Table 2:** Square root coefficients (RECM)

Technique	RECM
Kriging ordinary	0.814
Weiughted inverse of distance	0.843
Spline regular	4.937
Spline in tension	1.237

### Splines in tension

**Fig. 7:**
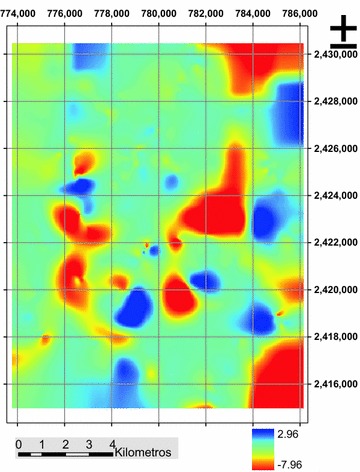
Estimation hard layer depth (m), Splines technique in tension

The estimate generated by this technique has less erratic values, but still has some positive values (Fig. 7). For RMSE, this technique had the second highest value of 1.237 (Table 2) so the relief representation of the hard layer was acceptable either.

### Weighted inverse technique

**Fig. 8:**
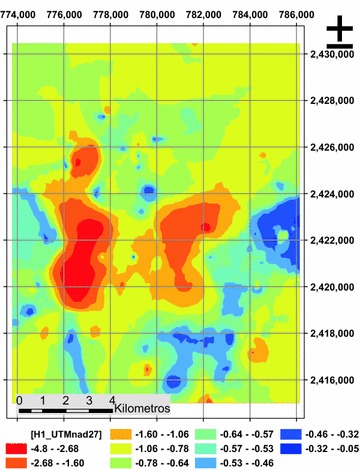
Estimated depth of hardpan (m) Technical inverse distance weighted

The estimate generated by the technique of Inverse weighted distance presents quite acceptable values at first, since they fail to generate positive (Fig. 8), but from the RMSE a value of 0.843 was obtained (Table 2), which is the third highest value, so, despite being more accurate than the two other techniques, Splines does not offer an accurate representation of the hard layer.

### Standard Kriging

**Fig. 9:**
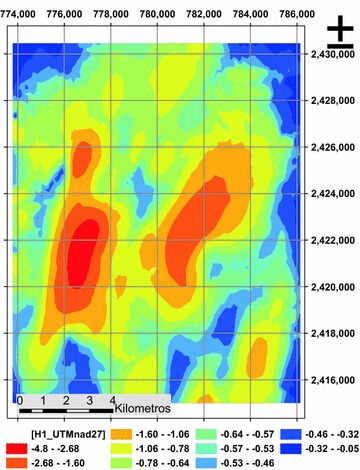
Estimating hardpan depth (m), ordinary Kriging technique

The estimate generated from ordinary Kriging technique has very acceptable values, as with the inverse distance weighted positive values are not generated, and as to the RMSE have the lowest value of all 0.814 (Table 2), so is the characterization technique that generated the relief of the hard layer more accurately (Fig. 9).

## Conclusions

The methodology used to evaluate the accuracy of different estimation techniques and spatial interpolation in order to decide which one provides the most accurate results.

The study shows that within deterministic methods to the case study, the technique of inverse distance weighted yielded the most accurate results, with a value of root mean square error (RMSE) of 0.843, while the technical regularized spline was the most inaccurate of all, with a RMSE of 4.937.

Noteworthy is the large difference between the RMSE values reported by regularized spline technique (RMSE = 4.937) and spline in tension (RMSE = 1.237).

The ordinary kriging estimator is the space that offered the most accurate results among the four techniques evaluated, with the lowest value of the root mean square error RMSE = 0.814.

These methods were chosen, among several techniques available in the literature, namely inverse weighted distance, regularized spline, tension spline and ordinary kriging, to estimate the depth of the hard layer of the Valley of Aguascalientes due to its popularity and robustness as interpolation methods for spatial prediction of variables in similar cases reported. Indeed, the technique of weighted inverse distance provides average values depending on the distance, perhaps this technique works better when the unknown points are closer to the sampling, so it is necessary to have a high density of known points. Regularized spline and tension spline tension interpolation functions, even when passing through the points of the model, are not able to predict the values of the checkpoints because of the roughness of the functions. These techniques provide good results when the variation of the surface is slight. Tension spline technique provides a closer approximation as modifies the minimization criterion incorporating the terms of the first derivative considering more abrupt variations without sacrificing the smoothness of the function. This technique is used when you want to preserve shape properties.

Ordinary Kriging technique, unlike the previous ones, uses random field theory to establish the spatial correlation of the variables achieving better prediction with a stochastic model. The advantage of the Kriging is that ensures that the average errors is zero. In ordinary Kriging, local media should not necessarily resemble the average of the entire population, so just take the closest points to make the interpolation, so the variables are correlated locally and not with the entire population. For this reason, ordinary Kriging is widely used for the prediction of variables whose value depends less on all samples collected, but the nearest properties such as geographic regions samples.

The hard layer depth in the valley of Aguascalientes has a clear spatial structure, capable of being modeled by adjusting variograms for estimation with ordinary Kriging technique.

Generally, it is recommended for the prediction of properties of clearly defined geographical areas, where properties to predict not depend greatly from the distant properties, but local, such as processes of formation of soil, deposition sequences and climatic focalized actions, so ordinary Kriging be preferred over other techniques.
